# New and traditional methods for antibiotic resistance genes removal: Constructed wetland technology and photocatalysis technology

**DOI:** 10.3389/fmicb.2022.1110793

**Published:** 2023-01-04

**Authors:** Pingping Chen, Xiaofei Yu, Jingyao Zhang, Yiqi Wang

**Affiliations:** State Environmental Protection Key Laboratory for Wetland Conservation and Vegetation Restoration & Jilin Provincial Key Laboratory of Ecological Restoration and Ecosystem Management & Key Laboratory of Vegetation Ecology of Ministry of Education, School of Environment, Northeast Normal University, Changchun, China

**Keywords:** antibiotic resistance genes, constructed wetlands, photocatalysis, removal mechanism, combination

## Abstract

Antibiotic resistance genes (ARGs) are a new environmental contaminant that poses a major hazard to humans and the environment. This research discusses the methods and drawbacks of two ARG removal approaches, constructed wetlands (CWs) and photocatalysis. CWs primarily rely on the synergistic effects of substrate adsorption, plant uptake, and microbial processes to remove ARGs. The removal of ARGs can be influenced by wetland plants, substrate type, wetland type, and hydraulic conditions. The absolute abundance of ARGs in effluent decreased, but their relative abundance increased. Photocatalysis deactivates ARGs predominantly through reactive oxygen species, with removal effectiveness determined by catalyst type, radiation type, and radiation intensity. The drawback is that it exposes intracellular resistance genes, perhaps increasing the risk of ARG spread. To address the current shortcomings, this paper proposes the feasibility of combining a constructed wetland with photocatalysis technology, which provides a novel strategy for ARG removal.

## 1. Introduction

Antibiotics have accumulated massively in the environment as a result of their widespread use in recent years. Antibiotics not only cause chemical pollution, but they may also induce the production of resistance genes (ARGs) and resistant bacteria (ARB) in the environment, hastening resistance spread and diffusion. As a result, the evolution and variation of bacterial resistance, as well as the spread of ARGs, have received increased attention in the field of environmental research. ARGs have been found in abundance in a variety of environmental media, including surface water, groundwater (Jiang et al., 2013), sediment, soil (Wang et al., 2019), and air detection. Antibiotic resistance has emerged as a serious global environmental health issue (Ying et al., 2017; Li et al., 2020). This antibiotic resistance can be spread between microorganisms *via* a horizontal gene transfer (HGT) mechanism (Berendonk et al., 2015). ARGs are classified as extracellular ARGs (eARGs) and intracellular ARGs (iARGs), both of which are transmitted *via* HGT.

ARGs enter the environment *via* a variety of routes, including municipal water, sewer runoff, livestock wastewater, landfill leachate, and hospital wastewater (Ezeuko et al., 2021; Koch et al., 2021). Antibiotics leave significant levels of ARGs in humans and animals, which are eventually discharged into wastewater treatment plants *via* fecal wastewater runoff. The wastewater discharged from wastewater treatment plants, as well as the ARGs present in biosolids, enter the soil and aquatic environments and are absorbed in a cycle by plants, animals, and so on. ARGs have been found in a range of water environments, and some investigations have revealed that they are present in tap water (Bergeron et al., 2015). ARG removal solutions that are cost-effective are urgently needed, and ARGs pollution must be addressed.

Some of the current tactics for removing ARGs from wastewater include disinfection procedures, membrane treatment technologies, advanced oxidation technologies, and constructed wetlands (CWs). Disinfection techniques, such as chlorine disinfection, which increases antibiotic resistance and the genera of bacteria that can carry antibiotic resistance, are ineffective in eliminating ARGs and also encourage the transmission and spread of ARGs (Cheng et al., 2021). ARGs are physically removed by membrane treatment technologies; nevertheless, when membrane filtration is used, ARGs accumulate in membrane fouling and sewage sludge, which can re-enter the environment. Advanced oxidation technologies and CWs, to the contrary hand, have relatively good ARG removal. ARGs can be effectively eliminated by photocatalytic advanced oxidation based on hydroxyl radicals, and a TiO_2_/UV treatment can reduce 5.8 log of *mecA* and 4.7 log of *ampC* (Guo et al., 2017). Furthermore, CWs is an efficient and sustainable wastewater treatment technology that efficiently removes organic matter, bacteria, antibiotics, pharmaceuticals and personal care products (PPCPs) from wastewater (Hartl et al., 2021), and has great potential in ARGs removal (Chen et al., 2016a; Huang et al., 2017). Thus, this study summarizes recent research and uses of CWs and photocatalysis in the removal of ARGs. The basic mechanisms, affecting factors, and limitations of CWs and photocatalysis for ARG removal are summarized. The feasibility of using CWs in conjunction with photocatalysis to remove ARGs is considered. Some novel approaches to removing ARGs from aquatic environments are proposed.

## 2. CWs for the removal of ARGs

**Figure 1:**
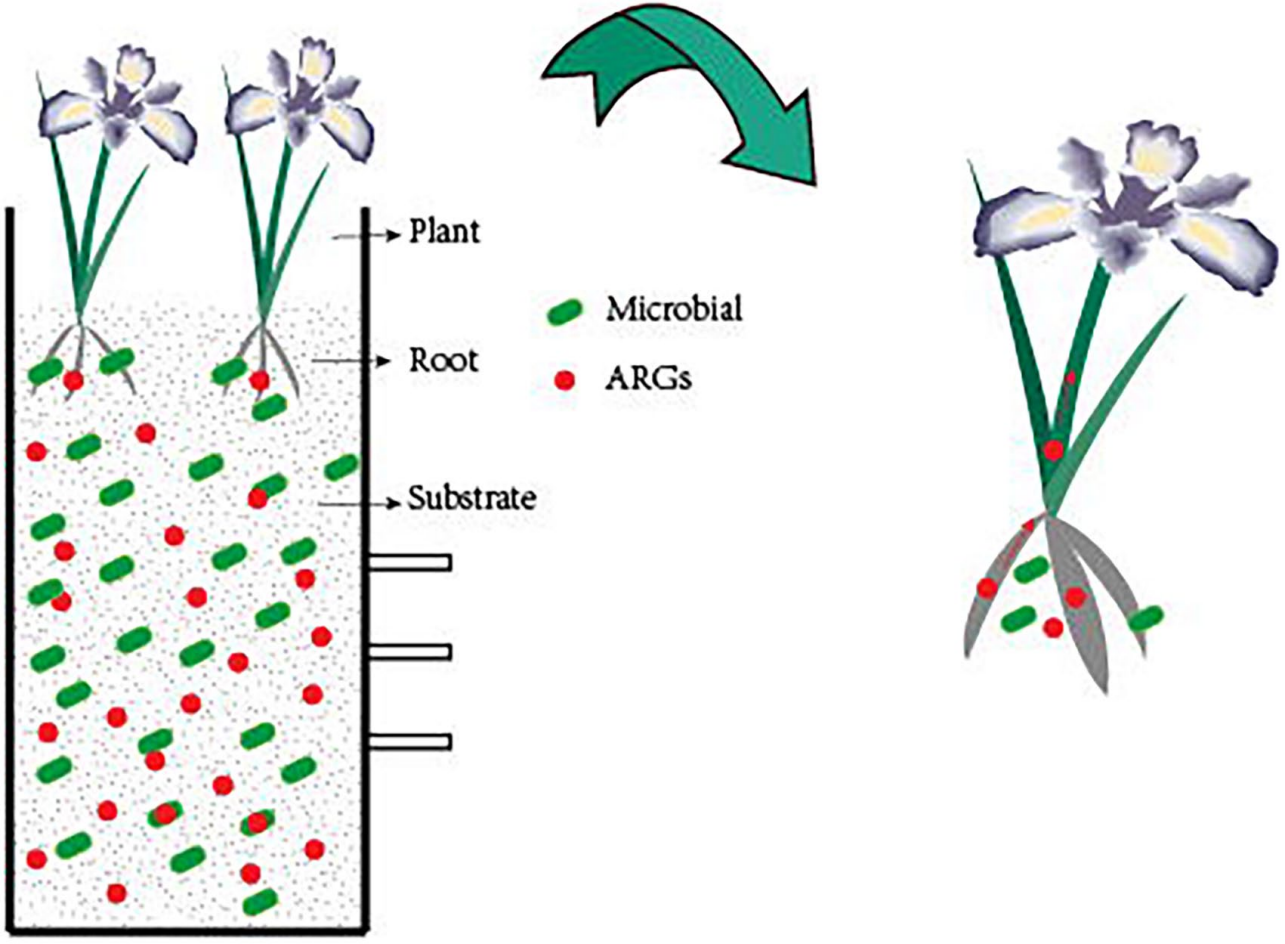
Mechanisms for the removal of ARGs in CWs. The substrate can absorb a high amount of ARGs, while the plant can also absorb ARGs. As the plant grows, ARGs migrate from the roots to the stems and leaves, and the plant root system’s biofilm structure removes ARGs by mechanisms of filtration, adsorption, absorption, and transformation of ARGs, and bacteria within the CWs can also remove ARGs.

CWs are an ecosystem made up of water, microbial communities, plants, and substrate (Chen et al., 2019). It uses a synergistic process of physical, chemical, and biological processes to remove contaminants. Its advantages over conventional wastewater treatment technologies include low cost, simplicity of use, and good maintenance (Faulwetter et al., 2009). CWs are currently being utilized to treat domestic wastewater (Adrados et al., 2014), agricultural wastewater (Liu J. et al., 2013), and landfill leachate (Sundberg et al., 2007). CWs are effective in removing both new pollutants as well as conventional contaminants like nitrogen and phosphorus. CWs remove ARGs mainly by substrate sorption, plant uptake, and microbial removal (Figure. 1). The type of wetlands, the plants used, the type of substrate, and other factors all have an impact on how successfully CWs remove ARGs. ARGs can currently be removed from aquatic habitats using CWs, albeit the bulk of these techniques are still in the experimental stage and the precise process is unknown, needing additional research.

### 2.1. Removal efficiency of ARGs for different CWs types

Surface flow constructed wetlands (SFCWs) and subsurface flow CWs are the two types of CWs. Subsurface flow CWs are classified as either horizontal subsurface flow constructed wetlands (HFCWs) or vertical subsurface flow constructed wetlands (VSFWs) based on the direction of the water flow (VFCWs). The removal effectiveness of ARGs varied dramatically amongst CW types (Table 1).

**Table 1:** ARGs removal effects in different CWs.

CW types	Target ARGs	Removal efficiency (%)	References
SFCWs	*sul1*, *sul2*, *tetG*, *floR*	47.2–82.8	Chen et al. (2016b)
SFCWs	*sul1*, *sul2*, *tetG tetM*, *qnrB*, *qnrS*	59.5–77.8	Fang et al. (2017)
HFCWs	*sul1*, *sul2*, *tetG*, *floR*	59.3–90.5	Chen et al. (2016b)
HFCWs	*sul1*, *tetW*, *tetG*, *dfrA1*, *aphA*, *tetX*, *ermC*, *tetO*	14.5–94.1	Du et al. (2021)
VFCWs	*sul1*, *sul2*, *tetG*, *floR*	79.1–94.6	Chen et al. (2016b)
VFCWs	*tetM*, *tetO*, *tetW*	90	Liu L. et al. (2013)
VFCWs	*tetO*, *tetM*, *tetW*, *tetA*, *tetX*, *intI1*	45.9–99.9	Huang et al. (2014)
VFCWs	*tetW*, *tetA*, *tetX*, *intI1*	33.2–99.1	Huang et al. (2017)

CWs have the ability to remove most common ARGs, with removal rates ranging from 14.5% to 100%. Furthermore, the ARG removal efficiency of several CWs was much higher than that of conventional wastewater treatment plants (Xu et al., 2015). Various types of CWs have their own targeted ARG species that are efficiently removed. For example, VFCWs significantly reduced the concentration of tetracycline resistance genes in livestock effluent (Liu L. et al., 2013), with the absolute abundance of *tetM*, *tetW*, and *tetO* being reduced by 90%. HFCWs was also effective in removing ARGs, especially sulfonamide ARGs. The abundance of *sul1*, *sul2*, *tetM*, *tetO*, *tetQ*, *tetW*, and *intI1* was reduced by 1–3 orders of magnitude by HFCWs (Chen and Zhang, 2013a). HFCWs was more effective in eliminating sul1-carrying bacteria than some typical wastewater treatment facilities (Czekalski et al., 2012), indicating that it can be used as a supplement to standard wastewater treatment plants, particularly to reduce the amount of sulfonamide ARGs. HFCWs and VFCW are more effective at removing contaminants than SFCWs, with VFCWs having the highest removal efficacy, ranging from 33.2 to 99.9%. The differences in removal effectiveness may be connected to the adsorptive filtration, biochemical processes, and redox conditions found in each wetland. Furthermore, the direction of water flow in VFCWs induced disparities in removal results, with upflow VFCWs having a higher relative abundance of tetracycline ARGs and *intI1* than downflow VFCWs (Chen et al., 2019).

### 2.2. ARGs uptake by plants in CWs and biofilm degradation in the root system

ARGs are primarily removed by plants in CWs *via* two pathways: absorption and root biofilm breakdown. Bacteria can reproduce in plant tissues and expand their populations during plant growth *via* hydraulic transport and active plant absorption. This allows plants to effectively reduce the abundance of microorganisms in the feed water, thus facilitating the removal of ARGs (Vacca et al., 2005). There were significant differences in the distribution of ARGs among different tissues of the plant, with ARG abundance higher in plant leaves than stems (Guo et al., 2021). The total abundance of ARGs observed in plants, however, was much lower than that found in the substrate.

The huge root systems of wetland plants can form a special biofilm structure together with the filler surface to remove ARGs through the processes of filtration, adsorption, absorption, and transformation of ARGs. Tetracycline ARGs, especially *tetW*, can rapidly migrate to the biofilm surface and are therefore more easily removed by CWs (Cheng et al., 2013). Furthermore, it has been demonstrated that plants can indirectly participate in the removal of ARGs, primarily by filtering solid particles and delivering oxygen to the microbial community, enhancing the role of inter-rhizosphere bacteria, or providing a medium for biofilm development, which improves the removal capacity of microorganisms and reduces the accumulation of ARGs, resulting in a reduction in ARGs abundance (Anderson et al., 2013; Fang et al., 2017). The type and amount of inter-root secretions vary among plants, thus affecting the inter-root microorganisms, resulting in different removal efficiencies of ARGs by different plants, with *Thalia dealbata* Fraser being more effective than *Iris tectorum* Maxim in removing ARGs (Chen et al., 2016b). Reed is a major aquatic plant for reducing ARGs contamination, with a removal effectiveness of more than 90% for ARGs such as *sul1*, *sul2*, *ermB*, *qnrS*, and *bla_TEM-1_* (Avila et al., 2021). However, biological processes in CWs not only degrade ARGs as described above, but also lead to the transfer and increase of ARGs (Ghosh and LaPara, 2007; Diehl and LaPara, 2010; Guo et al., 2014; Yang et al., 2014), thus the role in ARGs removal is complex and needs to be studied in more depth.

### 2.3. Substrate effect on ARGs removal

In CWs, the matrix serves as an essential vehicle for physicochemical processes (Chen et al., 2019). The particle size distribution, surface charge, porosity, and pH of the matrix all have an impact on ARGs removal (He et al., 2021). Substrate adsorption and microbial degradation on the substrate surface are two important pathways for ARG removal. The inadequate elimination of macrolide resistance genes by CWs may be explained by substrate adsorption (Chen et al., 2019). Bacteria, particularly gut microorganisms, are easily absorbed by substrates (Huang et al., 2017). Furthermore, because ARGs are generally carried by gut bacteria, the high removal effectiveness of ARGs by CWs is most likely due to the substrate’s high adsorption efficiency on intestinal microorganisms (Huang et al., 2014). ARGs from *tenericutes*, *cyanobacteria*, and *acidobacteria* were more likely to be lost (Su et al., 2019). In addition, small pore size substrates enable bacterial filtration and precipitation and have a great ability to remove microorganisms from water. Gravel, zeolite, oyster shell, medicinal stone, ceramic, and tuff are the most typical substrates used in CWs for ARGs removal (Table 2). Zeolites have a microporous structure and silica hydroxyl groups, which provide surface area for chemisorption and microbial attachment, while silica hydroxyl groups are catalytically active for various chemical reactions, and their average pore size (4.32 nm) is smaller than that of volcanic rocks (10.78 nm; Liu L. et al., 2013), making zeolites more efficient for the removal of ARGs (Gorra et al., 2007). Ceramics have a porous morphology with a greater specific surface area, but they have a macroporous structure (Chen et al., 2016a), hence they are less effective at removing ARGs than zeolites. Tuff has a more porous structure and a bigger surface area, allowing for more bacterial adsorption and stronger biofilm development (Abou-Kandil et al., 2021), and hence has a better ability to remove ARGs. Both oyster shell and medical stone have an ordered lamellar structure, but oyster shell has considerable agglomeration that medical stone does not, and hence oyster shell is more efficient at removing ARGs than medical stone.

**Table 2:** Comparison of adsorption performance of CWs substrate.

CWs	CWs substrates	Adsorption performance	References
VFCWs	Tuff, gravel	Tuff > gravel	Abou-Kandil et al. (2021)
VFCWs	Oyster shell, zeolite, medical stone, ceramic	Zeolite > oyster shell > medical stone > ceramic	Chen et al. (2016a)
VFCWs	Zeolite, volcanic rocks	Zeolite > volcanic rocks	Liu J. et al. (2013)

### 2.4. CWs remove the shortcomings of ARGs

The mechanism of ARG removal by CWs is complex, and the mechanism of migration and removal of ARGs in CWs is not well understood. While CWs are effective in removing ARGs, they also have the risk of enriching them. Despite a decrease in absolute abundance, the relative abundance of resistance genes in CWs effluent increased (Yi et al., 2017). ARGs are generally transmitted *via* vertical and horizontal gene transfer, i.e., genetic transfer between parents and transmission between microbes. Once ARGs produce resistance in pathogenic microbes or spread across pathogenic microorganisms, CWs can evolve into a large reservoir of ARGs, with potentially disastrous ecological and health effects. According to research, reducing the overall number of microorganisms in wastewater can successfully limit ARGs transmission (Song et al., 2018). Therefore, research on the removal of ARGs by CWs still faces a great challenge.

## 3. Photocatalytic removal of ARGs

Photocatalytic oxidation is a method of removing pollutants from water or the atmosphere that involves a sequence of reactions between a catalyst and oxygen in solution that produce powerful oxidizing ·OH under the action of solar radiation. The photocatalyst is central to photocatalytic technology, and there are many different types of photocatalysts, including TiO_2_, ZnO, and WO_3_, which are now the most researched photocatalysts. Because of its non-toxicity, low cost, and great photocatalytic efficiency, TiO_2_ is the most often used photocatalyst.

In comparison to the activated sludge method, membrane separation method, and chemical oxidation method, the photocatalytic oxidation method has the advantages of low energy consumption, rapid reaction, simple operation, and no secondary pollution, and it has become a popular research direction in recent years. Advanced photocatalytic oxidation processes have the potential to remove microbial contaminants, such as photocatalytic titanium dioxide, which can generate reactive oxygen species (ROS, e.g., OH), which kill microorganisms by oxidative damage to cell membranes, RNA, DNA, proteins, and lipids (Umar et al., 2019). TiO_2_ multiphase photocatalytic oxidation offers various advantages as a “green” water disinfection technology, including better efficiency in eliminating ARB from wastewater than standard disinfection processes and the absence of disinfection by-products.

### 3.1. Mechanism of photocatalytic removal of ARGs

**Figure 2:**
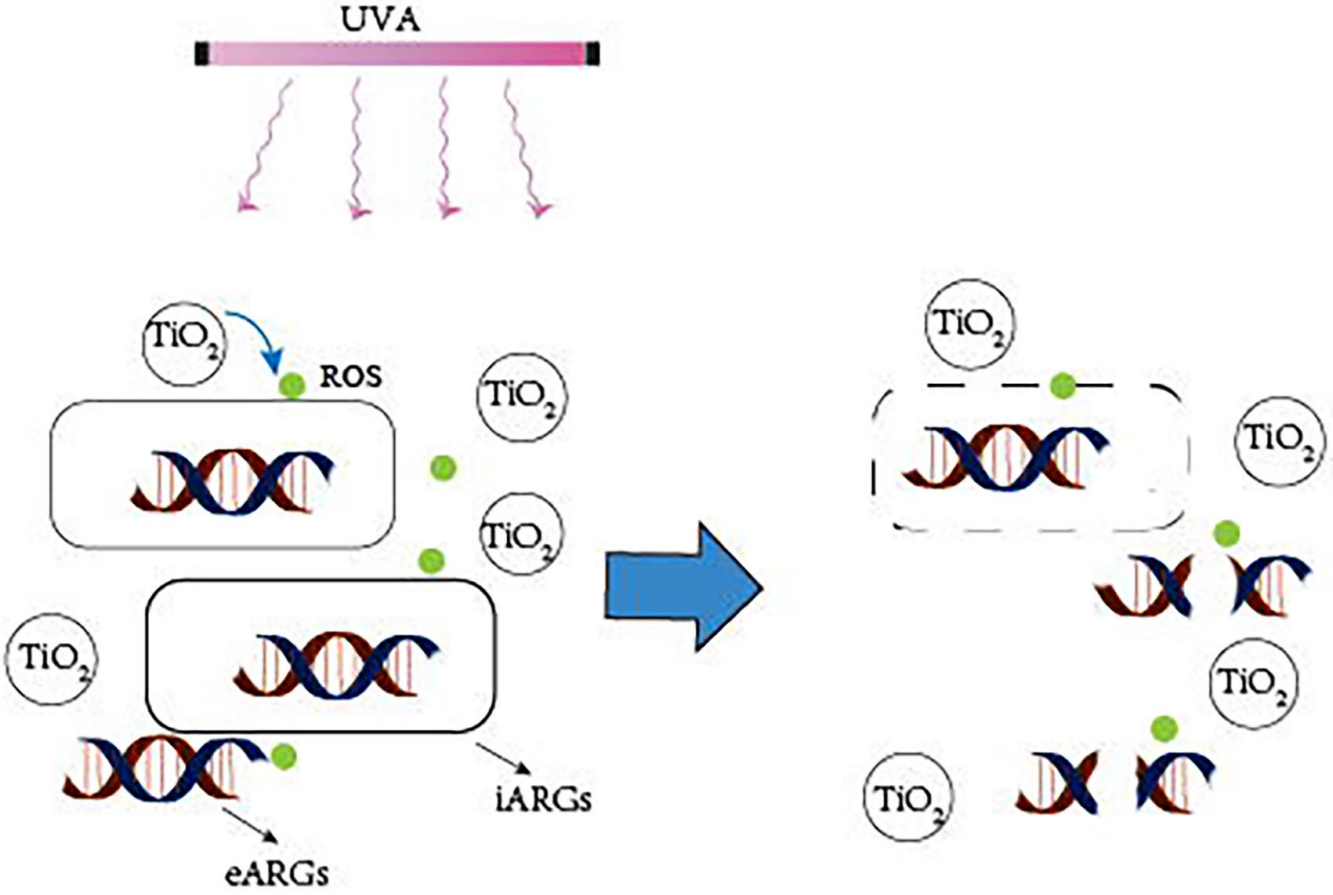
Mechanism of photocatalytic removal of ARGs. UVA irradiation causes the catalyst to create ROS, which attack the bacterial cell wall, breaking it down and converting iARGs to eARGs. Simultaneously, ROS attack eARGs, breaking the DNA strand, and inactivating ARGs.

Photocatalytic creation of holes and electrons under UVA irradiation causes redox processes in which oxidants first target microbial cell walls, membranes, and enzymes, and later interior components such as RNA and DNA (Koivunen and Heinonen-Tanski, 2005). Although bacteria have self-defense systems to defend themselves from ROS damage, excessive ROS can cause oxidative stress and assault membrane lipids, eventually leading to DNA damage (Li et al., 2011). ARG abundance is decreasing owing to photocatalyst light exposure, which causes DNA damage in bacterial cells (Figure 2). Furthermore, photocatalytic treatment breaks down long DNA strands into shorter nucleotides, allowing the deoxyribose phosphate backbone to be broken (Hirakawa et al., 2009). Free ARGs-containing deoxyribonucleic acids are due to a lack of protective bacterial cell walls, which can also be rapidly removed by photocatalysis. TiO_2_ can produce hydroxyl radicals, which are thought to be the most active oxidants for destroying ARGs (Pham and Lee, 2014). Therefore, the majority of current research employs TiO_2_ or modified TiO_2_ as a photocatalyst.

### 3.2. Factors influencing photocatalytic removal of ARGs

Among the large number of photocatalytic materials, TiO_2_ and graphitic carbon nitride (g-C_3_N_4_) are two materials that have been studied more. The effects of different catalyst types, radiation types, and radiation intensities on the removal of ARGs varied significantly (Table 3). Radiation intensity is significant in photocatalysis efficiency (Yu et al., 2020), and the higher the radiation intensity, the more effective the removal of ARGs. UV removal of ARGs is more effective than solar radiation, and the decrease of ARGs by TiO_2_ under UV irradiation is 4–5 log, whereas it is only 0.5 log under sunlight irradiation, which may be related to TiO_2_’s low activation effectiveness under sunshine. To address TiO_2_’s low activation efficiency in solar radiation, the synthesis and application of semiconductor-reinforced TiO_2_ composites have emerged as a hot research area. TiO_2_-rGO composites are found to be much more effective than TiO_2_ at removing ARGs. The removal effect of different ARGs under the same treatment conditions is different, most likely because their sensitivity to oxidation radicals varies depending on their composition. Because reactive oxygen species react more quickly with guanine bases in DNA, ARGs with a high GC% content, such as sulfonamide ARGs, are degraded to a greater extent (Ren et al., 2018). In addition, the amount of photocatalyst used also affects the removal of ARGs, and the size of the composite also influences the treatment efficacy, as smaller composites are more likely to cross cell membranes and promote rupture (Guo and Tian, 2019). Hence, the development of non-toxic nanocomposites should be explored in the development of new photocatalysts for the removal of ARGs. Inorganic ions and organic debris in wastewater can both limit photocatalytic activity by adsorbing on the TiO_2_ surface and blocking active sites, and some anions can function as cavity scavengers, slowing the rate of chemical breakdown or disinfection on the TiO_2_ surface. In addition, organic matter in wastewater can also reduce the disinfection rate through a variety of mechanisms, including ROS scavenging, competition for active sites on the TiO_2_ surface, and direct UV absorption.

**Table 3:** Effect of different catalysts, radiation type and radiation intensity on the removal effectivness of ARGs.

Catalyst	Radiation type	Radiation intensity	ARGs	ARGs removal (log units)	References
TiO_2_	UVA	120 mJ/cm^2^	*ampC*, *mecA*	4.7log (*ampC*), 5.8log (*mecA*)	Guo et al. (2017)
TiO_2_	UVA	8 W/m^2^	*Bla_NDM-1_*	0.7–1.5log	Chen et al. (2022)
TiO_2_	Solar simulator	500 W/m^2^	*sul1*, *sul2*, *bla_TEM_*, *int11*, *uidA*, *efec*	98.9% (*sul1*), 74.6% (*sul2*), 93.26% (*bla_TEM_*), 93.45% (*int11*),99.96% (*uidA*),71.96% (*efec*)	Felis et al. (2022)
TiO_2_, TiO_2_-rGO	Solar simulator	63 W/m^2^	*ampC*, *sul1*, *ermb*, *mecA*	2log *ampC* (TiO_2_-rGO); 0.5 log *ermB* (TiO_2_)	Karaolia et al. (2018)
Ag/AgBr/g-C_3_N_4_	UVA	9. 6 W/m^2^	*tetA, tetM*, *tetQ*, *intI1*	49% (*tetA*), 86% (*tetM*), 69% (*tetQ*), 86% (*intI1*)	Yu et al. (2020)
g-C_3_N_4_	UVA	3 W/m^2^	*tetA*, *tetB*,	41.77% (*tetA*), 37.59% (*tetB*)	Hu et al. (2022)
TiO_2_, GO-TiO_2_	Solar radiation	40 W/m^2^	*int11*, *qnrS*, *bla*_*CTK*__-M_, *sul1*	3.5 log *bla*_*CTK*__-M_ (GO-TiO_2_)	Moreira et al. (2018)

### 3.3. Effect of nanomaterials on the diffusion of ARGs

The effect of nanomaterials on the HGT of ARGs in the environment has aroused the interest of many researchers. Related research has revealed that gene level transfer is a significant factor in the spread of ARGs (Cheng et al., 2021). ARGs can move from one bacterium to another when combined with mobile genetic elements like plasmids, integrons, transposons, and so on, allowing bacteria to acquire ARGs. Most nanomaterials can contribute to the diffusion of ARGs in pure bacterial systems. Nano-TiO_2_ significantly improved the splice transfer between RP4 plasmids in *E. coli* (Qiu et al., 2015), and with slight inhibition of bacterial growth, it could increase the splice transfer efficiency by 56-fold. ARB activity was reduced by graphene oxide (GO). However, at the level of ARGs, all GO greatly boosted transfer efficiency (Guo and Zhang, 2017). These findings suggest that the excellent adsorption characteristics of insoluble nanoparticles boost the binding of ARGs and bacteria, which can considerably contribute to the transfer efficiency of ARGs. The environmental conditions in actual water and wastewater treatment are complex, as are the technologies used. The environmental conditions in actual water and wastewater treatment are complicated, with a generally mixed flora of microorganisms. The impact of nanomaterials on the spread of ARGs in the actual world is more complicated. The effluent from a secondary wastewater treatment plant was treated using polyvinylidene fluoride ultrafiltration membranes enhanced with nano-TiO_2_ (Ren et al., 2018). The results showed that nano-TiO_2_ on this membrane was successful in eliminating 98% of ARGs when exposed to UV light, thus effectively controlling the HGT of ARGs. To remove resistant bacteria and genes from municipal wastewater, TiO_2_-rGO material was utilized (Karaolia et al., 2018). These results can be attributed to the ability of the reactive oxygen species generated by photoexcitation of nanomaterials to oxidatively damage DNA, thus enabling the control of the proliferation of ARGs in the real environment.

### 3.4. Photocatalysis’ shortcomings in ARG removal

Photocatalysis is an excellent method for removing ARGs by directly destroying cellular deoxyribonucleic acid; however, the continuous use of high-intensity UV light during wastewater treatment is difficult (Chen and Zhang, 2013b; Lee et al., 2017; Mauter et al., 2018). Moreover, after UV damage to the cell wall, iARGs flow out and are converted into eARGs, which can survive in the environment and bind to other bacteria *via* transformation, transduction, and other mechanisms, resulting in the spread of ARGs in the environment. In addition, genes fragmented by oxidation can integrate with other pathogens in the wastewater (De Vries and Wackernagel, 2002; Nielsen et al., 2007), thus requiring additional treatment. Bacteria with ruptured cell membranes can cause cell lysis and an increase in eARGs levels, resulting in secondary water contamination. The removal of ARGs released by ARBs is critical. Furthermore, injured bacteria treated with photocatalysis will recover, and in addition to bacterial regrowth, ARGs transfer may rise if pathogens are not completely inactivated. The release and transfer of ARGs may contribute to the subsequent development of resistant bacteria in the aquatic environment unless the duration of treatment is managed (Dunlop et al., 2015).

## 4. Prospect

CWs can efficiently remove ARGs, and the removal efficiency of VFCWs and HFCWs is greater than that of SFCWs. VFCWs effectively remove tetracycline ARGs, whereas HFCWs effectively remove sulfonamide ARGs. Furthermore， photocatalysis successfully removes ARGs, and the higher the intensity of the irradiation, the better the efficacy of ARGs removal. For the elimination of ARGs, nanocomposites outperformed TiO_2_. Combining the advantages of CWs and photocatalysis in the removal of ARGs, a combination of CWs and photocatalysis could be proposed to remove ARGs. With remarkable success, the combination of photocatalysis and CWs has been studied for the treatment of high-salt chromium-containing wastewater (Li et al., 2020) and municipal wastewater. More importantly, in the combination system of CWs and photocatalysis, CWs could further eliminate the eARGs. Under UVA irradiation, the catalyst generates ROS, and the ROS break the cell wall and allow intracellular DNA to flow out, converting the difficult-to-remove iARGs into eARGs. While the ROS also attacks the eARGs, breaking the DNA strands and inactivating the ARGs. eARGs and iARGs are removed further in the artificial wetland by substrate adsorption, plant uptake, and microbial action. Furthermore, the combination would be increased the removal efficiency of COD, BOD_5_, and Cr (VI) with no negative effect on plant production indicators (Li et al., 2019). In addition, photocatalysis can extend the life of the wetland because it eliminates clogging with refractory substances, reduces total phosphorus concentrations and reduces wetland loading.

Therefore, CWs paired with photocatalytic wastewater treatment technology provide the advantages of steady water quality, minimal investment, and low running costs. When CWs are combined with photocatalytic technology, they may remove both iARGs and eARGs, and the treatment is complete, lowering the risk of ARGs spreading. Moverover, CWs in combination with photocatalysis could produce a flexible and operable wastewater treatment system. This method combines ecological treatment technology with photocatalysis technology to produce a new technology that is superior to traditional wastewater treatment methods to remove resistance genes.

## Author contributions

PC: data curation and analysis, writing—original draft. XY: worked on the technical details, supervised the findings of the work and helped in the development of manuscript. JZ: aided in interpreting the results and worked on the manuscript. YW: writing—review and editing. All authors contributed to the article and approved the submitted version.

## Funding

This study was supported by the National Natural Science Foundation of China (42222102).

## Conflict of interest

The authors declare that they have no known competing financial interests or personal relationships that could have appeared to influence the work reported in this paper.

## Publisher’s note

All claims expressed in this article are solely those of the authors and do not necessarily represent those of their affiliated organizations, or those of the publisher, the editors and the reviewers. Any product that may be evaluated in this article, or claim that may be made by its manufacturer, is not guaranteed or endorsed by the publisher.
